# Differential Depletion of Bone Marrow Resident B-ALL after Systemic Administration of Endosomal TLR Agonists

**DOI:** 10.3390/cancers12010169

**Published:** 2020-01-10

**Authors:** Sumin Jo, Abbas Fotovati, Jesus Duque-Afonso, Michael L. Cleary, Peter van den Elzen, Alix E. Seif, Gregor S.D. Reid

**Affiliations:** 1 Michael Cuccione Childhood Cancer Research Program, BC Children’s Hospital Research Institute, Vancouver, BC V5Z 4H4, Canada; suminjo@alumni.ubc.ca (S.J.); fotovati@mail.ubc.ca (A.F.); 2 Department of Pathology, School of Medicine, Stanford University, Stanford, CA 94305, USA; jesus.duque.afonso@uniklinik-freiburg.de (J.D.-A.); mcleary@stanford.edu (M.L.C.); 3 Department of Pathology and Laboratory Medicine, University of British Columbia, Vancouver, BC V6T 1Z4, Canada; pvde@mail.ubc.ca; 4 Abramson Family Cancer Research Institute, University of Pennsylvania, Philadelphia, PA 19104, USA; seifa@email.chop.edu; 5 Department of Pediatrics, University of British Columbia, Vancouver, BC V6T 1Z4, Canada

**Keywords:** acute lymphoblastic leukemia, minimal residual disease, bone marrow, toll-like receptor, innate immunity, immunotherapy

## Abstract

Acute lymphoblastic leukemia (ALL) is the most common pediatric malignancy. While frontline chemotherapy regimens are generally very effective, the prognosis for patients whose leukemia returns remains poor. The presence of measurable residual disease (MRD) in bone marrow at the completion of induction therapy is the strongest predictor of relapse, suggesting that strategies to eliminate the residual leukemic blasts from this niche could reduce the incidence of recurrence. We have previously reported that toll-like receptor (TLR) agonists achieve durable T cell-mediated protection in transplantable cell line-based models of B cell precursor leukemia (B-ALL). However, the successful application of TLR agonist therapy in an MRD setting would require the induction of anti-leukemic immune activity specifically in the bone marrow, a site of the chemotherapy-resistant leukemic blasts. In this study, we compare the organ-specific depletion of human and mouse primary B-ALL cells after systemic administration of endosomal TLR agonists. Despite comparable splenic responses, only the TLR9 agonist induced strong innate immune responses in the bone marrow and achieved a near-complete elimination of B-ALL cells. This pattern of response was associated with the most significantly prolonged disease-free survival. Overall, our findings identify innate immune activity in the bone marrow that is associated with durable TLR-induced protection against B-ALL outgrowth.

## 1. Introduction

Acute lymphoblastic leukemia (ALL) is the most common pediatric malignancy, with current long-term survival rates upwards of 80% in the developed world [1]. However, for the approximately 20% of children whose leukemia recurs, the prognosis is significantly worse and treatment options are limited [1,2]. As greater than 95% of patients with ALL achieve a complete remission with contemporary chemotherapy, a pragmatic approach to improving overall outcome is to reduce the incidence of recurrence by applying novel therapy during the remission stage to further deplete leukemic blasts.

The early bone marrow response to remission-induction chemotherapy is well recognized as a critical measure of initial treatment success [3,4]; the presence of measurable residual disease (MRD) in the bone marrow after this treatment phase is the most powerful predictor of relapse in ALL [5,6]. As a result, MRD status is a primary determinant of risk and informs treatment selection. While leukemia is subsequently eradicated from many MRD positive patients following intensified therapy, early MRD positivity is still associated with significantly poorer long-term outcome [7].

The absolute lymphocyte count (ALC) at the end of induction therapy is also reported to be a strong prognostic indicator in pediatric ALL; patients with high ALC are at significantly reduced risk of recurrence [8,9,10,11,12]. While the biology underlying this association remains unclear, we observed that the ALC was not simply a reflection of bone marrow recovery [13]; numbers of specific immune cell subsets, including T cells and dendritic cells, were most strongly correlated with ALC, suggesting a potential role for the endogenous immune system in the prevention of recurrence. Stimulation of this immune environment during early treatment could contribute to greater immune control of ALL progression.

Toll-like receptor (TLR) agonists can profoundly alter the tumour microenvironment, overcoming tolerance to tumour antigens and improving anticancer immunity without the need for increasing antigenicity by enhancing immunogenicity and/or the susceptibility to immune attack [14,15,16]. Despite limited clinical successes to date [17], preclinical studies continue to reinforce the development of TLR agonists for therapeutic use, particularly in combination with other agents [18,19,20,21]. Supporting a role for TLR stimulation as a strategy to eliminate residual ALL blasts by overcoming their weak T cell stimulatory capacity [22,23], we previously reported the ability of TLR agonists to enhance the immunogenicity of pediatric B cell precursor ALL (B-ALL) blasts [24,25]. Furthermore, treatment of ALL-bearing mice with CpG ODN, an agonist for TLR9, induced significant immune-mediated killing of human leukemia cells [26] and achieved durable T cell-dependent protection against outgrowth of transplanted syngeneic B-ALL cell lines [27].

The induction of protective immunity against ALL in a clinical MRD setting will require overcoming two significant obstacles: the location of treatment-resistant leukemic blasts in potentially immune-privileged niches, and the extremely low burden of neoantigens expressed by primary ALL blasts [28,29]. Given the ability of TLR9 signaling to confer protection against B-ALL [27], we compared a panel of endosomal TLR agonists for their ability to target the bone marrow niche and achieve protection against outgrowth of adoptively transferred primary B-ALL cells in both syngeneic mouse models and primary patient-derived xenografts (PDX).

## 2. Results

### 2.1. Direct and Indirect Cytotoxic Effects of Endosomal TLR Agonists on Mouse Primary B-ALL Cells In Vitro

**Figure 1:**
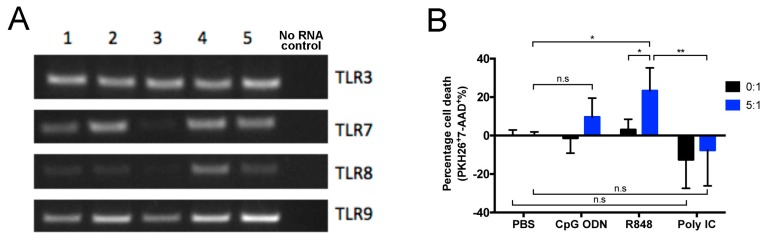
TLR expression and response by Eμ-ret mouse-derived primary leukemia cells. (**A**) mRNA from B-ALL cells from five leukemic Eμ-ret mice (1–5) was analyzed for the expression of transcripts for TLRs 3 and 7–9 by qualitative RT-PCR. (**B**) Cytotoxic effects of endosomal TLR stimulation on primary B-ALL cells cultured in the absence (0:1) or presence (5:1) of splenocytes from wild-type BALB/c mice. Results shown are pooled from four independent experiments. Two-way ANOVA with Tukey’s multiple comparisons test, bars represent mean ± SD; * *p* < 0.05, ** *p* < 0.01, n.s = not significant.

Eμ-ret mice, which express the RFP/RET fusion gene under transcriptional control of the immunoglobulin heavy chain enhancer, display an abnormally expanded late pro-B cell population at birth and succumb to B-ALL between 3 and 12 months of age [30,31]. To determine the potential of primary B-ALL to respond directly to endosomal TLR agonists, we evaluated leukemia cells from Eμ-ret mice with overt disease for expression of TLRs 3, 7, 8, and 9. All five independent leukemia samples tested expressed each TLR (Figure 1A). Despite expression of the relevant receptors, in 16-h cytotoxicity assays in which primary B-ALL cells were stimulated with CpG ODN (TLR9), R848 (TLR7), or polyI:C (TLR3) in the absence or presence of syngeneic splenocytes, each TLR agonist exerted only minimal direct cytotoxicity, as measured by changes in leukemia cell viability in the absence of immune effector cells (Figure 1B). In the presence of splenocytes, however, B-ALL viability was reduced but only achieved statistical significance with R848. As the degree of leukemia killing achieved was independent of TLR expression by B-ALL blasts, we next evaluated the influence of the immune microenvironment on TLR-induced anti-leukemia activity in vivo using a panel of primary Eμ-ret leukemia samples that were not characterized for TLR expression.

### 2.2. Organ-Specific Depletion of Primary B-ALL Cells by Endosomal TLR-Induced Innate Immune Responses

**Figure 2:**
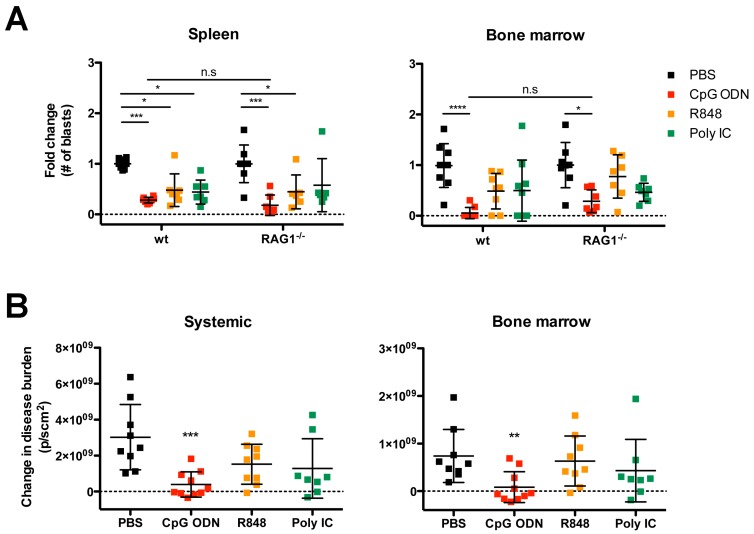
Endosomal TLR-induced immune activity is sufficient to initiate in vivo depletion of primary B-ALL. (**A**) Wild-type (wt) and RAG^−/−^ BALB/c mice bearing primary Eμ-ret B-ALL cells received a single 100 μg dose of indicated TLR agonists. Results shown are pooled from four independent experiments: PBS-treated wt (n = 8) and RAG^−/−^ BALB/c (n = 8); CpG ODN-treated wt (n = 8) and RAG^−/−^ BALB/c (n = 7); R848-treated wt (n = 7) and RAG^−/−^ BALB/c (n = 6); polyI:C-treated wt (n = 7) and RAG^−/−^ BALB/c (n = 6). (**B**) NOD/SCID mice bearing human primary ALL expressing firefly luciferase were treated with the indicated TLR agonists and disease burden measured systemically and in bone marrow by in vivo bioluminescence imaging. Results shown are pooled from four independent experiments. PBS-treated NOD/SCID (n = 9); CpG ODN-treated NOD/SCID (n = 10); R848-treated NOD/SCID (n = 9); polyI:C-treated NOD/SCID (n = 8). (**A**) Tukey’s multiple comparisons test, bars represent mean ± S.D.; * *p* < 0.05, *** *p* < 0.001, **** *p* < 0.0001, n.s = not significant. (**B**) Dunn’s multiple comparisons test, bars represent mean ± S.D.; ** *p* < 0.01, *** *p* < 0.001.

**Figure A1:**
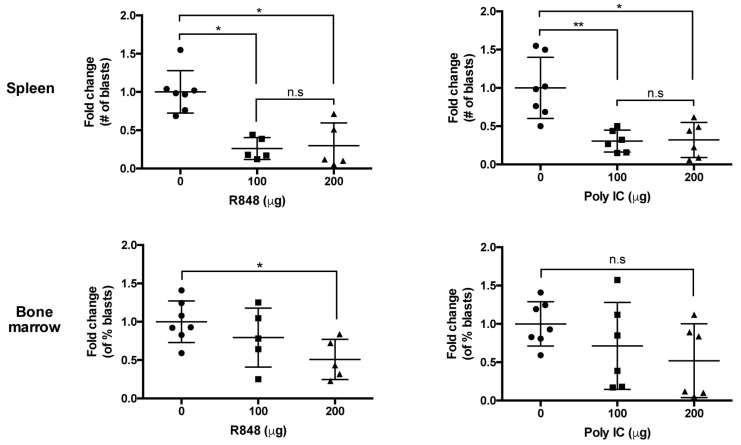
Comparison of dosage effects for TLR ligands. wt BALB/c mice bearing syngeneic primary B-ALL cells or 289 cell line received a single dose of indicated TLR agonists at 100 μg or 200 μg. Three days after the treatment, the disease burden in the spleen and bone marrow of polyI:C- and R848-treated mice were evaluated. Dunn’s multiple comparisons test, bars represent mean ± S.D. Results shown are pooled from two independent experiments; PBS-treated mice (n = 7); PolyI:C-treated mice (n = 6/dosage); R848-treated mice (n = 5/dosage) n.s = not significant.

The in vitro cytotoxicity assay revealed the differential capacity of endosomal TLR agonists to induce splenocyte-mediated killing of B-ALL. To determine whether this result was indicative of their ability to deplete B-ALL from in vivo niches, we evaluated early organ-specific changes in leukemia burden after systemic administration of endosomal TLR agonists to Eμ-ret B-ALL engrafted wild-type (wt) and RAG1^−/−^ (lacking T, B, and NKT cells) BALB/c mice. Seven days after injection of syngeneic primary B-ALL cells, by which time leukemia had engrafted the bone marrow [27] mice were randomized to receive a single 100 μg dose of CpG ODN, R848, polyI:C, or PBS intraperitoneally. Spleen and bone marrow were assessed for disease burden three days after treatment. The single dose of TLR agonist was sufficient to stimulate anti-ALL immune activity that depleted B-ALL cells in the spleen of both wt and RAG1^−/−^ mice (Figure 2A). In contrast to the in vitro assay (Figure 1), CpG ODN induced the strongest in vivo response in both strains. Furthermore, a reduction in leukemia burden in bone marrow was only achieved after CpG ODN treatment. While bone marrow depletion was greater in wt mice that possess full immune responsiveness, the reduction in B-ALL burden in RAG1^−/−^ mice reveals that a significant component of this activity is mediated by recombination-independent immune cell subsets (Figure 2A). A similar degree of leukemia depletion was observed using 200 μg of R848 and polyI:C (Figure A1, Appendix A), indicating that dosage is unlikely to explain the reduced efficacy of these agents.

To validate the potential clinical relevance of the immune stimulation profiles in diverse niches capable of supporting human B-ALL, we administered endosomal TLR agonists to NOD/SCID mice (which lack T, B, and NKT cells) xenografted with luciferase-tagged, patient-derived primary B-ALL [32]. When the average radiance from human ALL-engrafted mice reached ~1 × 10^7^ p/scm^2^, we randomly assigned mice to treatment groups receiving 3-dose regimens of individual TLR agonists (multiple doses were administered as leukemia burden was higher than in BALB/c mouse experiments). Four days after the last treatment, disease burden was evaluated systemically and in the bone marrow by bioluminescent imaging. While each TLR agonist reduced systemic disease burden compared to PBS-treated control mice, only the CpG ODN treatment achieved significant depletion of the primary human B-ALL systemically and in bone marrow (Figure 2B).

### 2.3. Immunostimulatory Effects of Endosomal TLR Agonists in Mice Bearing Primary B-ALL

**Figure 3:**
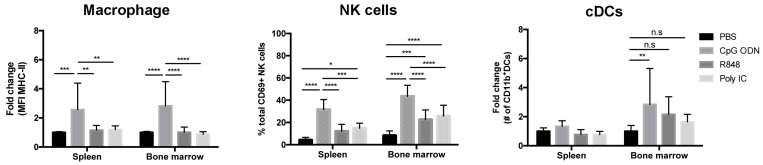
CpG ODN is sufficient to exert immunomodulatory effects in mice bearing B-ALL. Three days after a single-dose of endosomal TLR agonist, the percentage or number of activated innate immune cells in spleens and bone marrow of wt BALB/c mice bearing primary Eμ-ret B-ALL cells were measured. Graphs depict the fold change in the expression levels of MHC class II on macrophages (left panel), the percentage of activated NK cells among total viable NK cells (middle panel), and the fold change in the absolute number of CD11b^+^ cDCs (right panel). Results shown are pooled from four independent experiments: PBS-treated mice (n = 15); CpG ODN-treated mice (n = 14); R848-treated mice (n = 15); polyI:C-treated mice (n = 17). Dunn’s multiple comparisons tests, bars represent mean ± S.D.; * *p* < 0.05, ** *p* < 0.01, *** *p* < 0.001, **** *p* < 0.0001. n.s = not significant.

**Figure A2:**
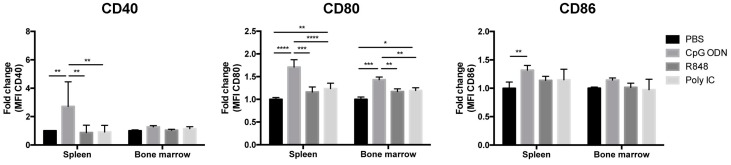
TLR-induced upregulation of co-stimulatory molecules on macrophages of mice bearing B-ALL. Graphs depict the fold change in the expression levels of CD40 (left panel), CD80 (middle panel) and CD86 (right panel) on macrophages in bone marrow and spleen were measured three days after a single-dose treatment with endosomal TLR agonist. The geometric MFI is shown. Tukey’s multiple comparisons test, bars represent mean ± S.D.; * *p* < 0.05, ** *p* < 0.01, *** *p* < 0.001, **** *p* < 0.0001. Results shown are pooled from four independent experiments; PBS-treated mice (n = 15); CpG ODN-treated mice (n = 14); R848-treated mice (n = 15); PolyI:C-treated mice (n = 17).

**Figure A3:**
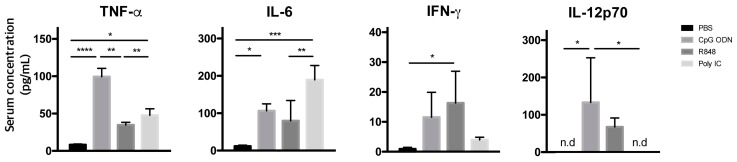
Evaluation of pro-inflammatory cytokine response induced by endosomal TLR agonists in mice bearing mouse primary leukemia. At day 7 after leukemia challenge, serum concentration of TNF-α, IL-6, IFN-γ, and IL-12 were measured 16 h after treatment in wt BALB/c mice bearing Eμ-ret B-ALL. Results are shown in averages of duplicate wells; n = 3 mice per group. Tukey’s multiple comparisons test, bars represent mean ± S.D.; * *p* < 0.05, ** *p* < 0.01, *** *p* < 0.001, **** *p* < 0.0001; n.d = not detected.

**Figure A4:**
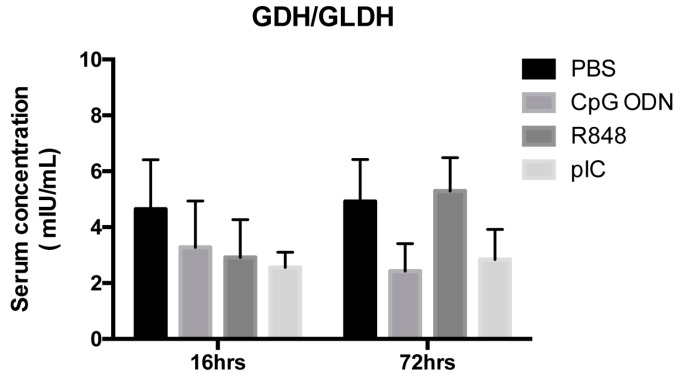
Detection of liver-specific serum enzyme GDH/GLDH in wt BALB/c mice following systemic administration of a single 100 μg dose of TLR agonist. Serum concentration of GDH/GLDH was measured 16 h and 72 h after treatment. Results are shown in averages of duplicate wells; n = 4 mice per group for PBS- and R848- treated and n = 5 mice per group for CpG ODN- and polyI:C-treated wt BALB/c.

We have previously reported that durable protection against B-ALL cell lines generated by CpG ODN is dependent on a CD4/CD8 T cell response [27]. To determine whether the endosomal TLR agonists differ not only in depletion activity but also in their capacity to stimulate immune cell subsets associated with priming antigen-specific immune responses, we evaluated cell populations in wt BALB/c mice bearing Eμ-ret B-ALL three days after systemic administration of a single dose of TLR ligand. Upregulation of activation markers on macrophages (F4/80^+^CD11b^+^CD11c^−^) was most significant in CpG ODN-treated mice, while expression of CD40 and CD80 (Figure A2, Appendix A) were only upregulated in the spleen; higher expression of MHC class II molecules (Figure 3) and CD86 (Figure A2, Appendix A) were detected in both spleen and bone marrow. Similarly, the strongest activation of NK cells (CD69^+^CD335^+^) was observed in both spleen and bone marrow of CpG ODN-treated mice (Figure 3). In addition, the number of CD11b^+^ cDCs (CD11c^+^B220^−^MHC-II^+^) was increased only in the bone marrow of CpG ODN-treated mice (Figure 3). Confirmation that the dosing used was sufficient to achieve systemic immunomodulation with each TLR agonist was provided by the significant, but distinct, increase in pro-inflammatory cytokines in the peripheral blood 16 h after treatment (Figure A3, Appendix A). Notably, systemic administration of TLR agonists at a sufficient dose to achieve immune cell activation, pro-inflammatory cytokine production, and B-ALL depletion was not accompanied by an increase in liver enzymes (GDH/GLDH) in serum that is indicative of acute hepatotoxicity (Figure A4, Appendix A).

### 2.4. Systemic Administration of TLR Agonists Achieves Durable Control of Primary B-ALL Progression In Vivo

**Figure 4:**
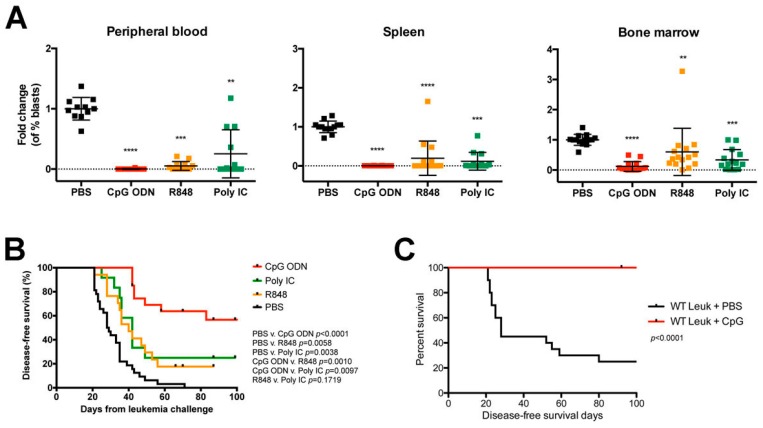
Endosomal TLR-mediated immune stimulation induces protective immune responses in leukemia-bearing mice. BALB/c mice engrafted with syngeneic primary Eμ-ret B-ALL cells were treated with indicated TLR agonists and (**A**) evaluated for disease burden in peripheral blood (left), spleen (middle), and bone marrow (right) six days after the last treatment, or (**B**) monitored for survival. Results shown are pooled from five independent experiments. PBS-treated (n = 11), median survival = 28.5 days; CpG ODN-treated (n = 16); R848-treated (n = 16), median survival = 40 days; polyI:C-treated (n = 16), median survival = 42 days. (**C**) CD1d-deficient BALB/c mice engrafted with CD1d+ primary Eμ-ret B-ALL cells and treated with PBS (n = 20) or CpG (n = 16). (A) Dunn’s multiple comparisons test, bars represent mean ± S.D. ** *p* < 0.01, *** *p* < 0.001, **** *p* < 0.0001. (B,C) Log-rank test.

**Figure A5:**
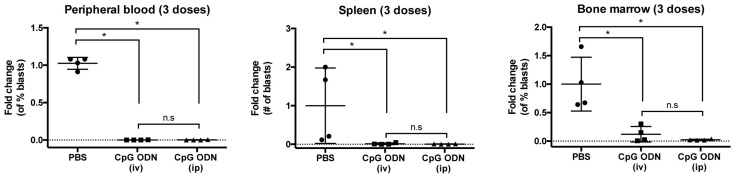
Systemic depletion of syngeneic primary B-ALL cells treated with three 100 μg doses of TLR9 agonist delivered intravenously (iv) or intraperitoneally (ip). Leukemia burden was evaluated six days after the last treatment. Mann-Whitney test, bars represent mean ± S.D.1; * *p* < 0.05. Results shown are from one independent experiment; n = 4 mice per group. n.s = not significant.

Having demonstrated the superior ability of CpG ODN to induce immune stimulation that depletes syngeneic primary B-ALL blasts in the bone marrow microenvironment, we evaluated whether this activity was associated with better long-term outcome. Using our established 3-dose treatment protocol [27], we administered each TLR agonist to BALB/c mice on day 7 after the B-ALL injection and assessed cohorts for organ burden six days after the last treatment or monitored for survival. Each TLR agonist reduced leukemia burden in both sites, as well as peripheral blood, but did so to varying degrees; consistent with the single-dose experiments, R848 or polyI:C treatments failed to match the near-complete elimination of B-ALL cells achieved with CpG ODN treatment, with the largest disparity in leukemia burden detected in bone marrow (Figure 4A). A comparable level of leukemia depletion was achieved when systemic CpG ODN delivery was performed intravenously (Figure A5, Appendix A). In line with our previous reports using B-ALL cell lines, CpG ODN treatment conferred a significant survival advantage against primary Eμ-ret B-ALL, such that over 50% of treated BALB/c mice maintained durable remissions (Figure 4B). In contrast, only a modest increase in disease-free survival was achieved for polyI:C- and R848-treated mice, with a median survival of 42 days and 40 days, respectively, compared to that of 28.5 days for PBS-treated control mice.

**Figure A6:**
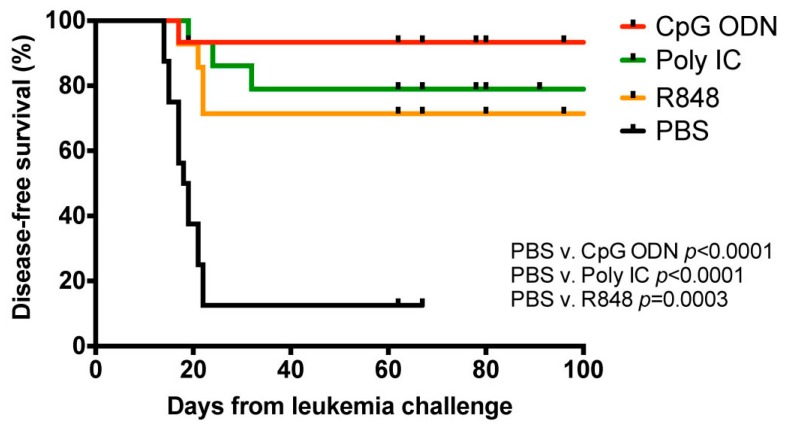
Endosomal TLR-mediated immune stimulations induce long-term protective immune responses in mice bearing E2A-PBX1 B-ALL. wt C57BL/6 mice bearing syngeneic primary E2A-PBX1 B-ALL cells treated with indicated TLR agonists were monitored and followed for survival. Results shown are pooled from five independent experiments. PBS-treated (n = 16), median survival = 18.5 days; CpG ODN-treated (n = 15); R848-treated (n = 14); PolyI:C-treated (n = 15). Log-rank test.

While significant, the CpG ODN-induced survival benefit achieved for BALB/c mice engrafted with primary B-ALL was lower than previously reported for those receiving the 289 B-ALL cell line [27]. Consistent with the hypothesis that the reduced efficacy of CpG ODN against primary ALL was a consequence of low antigen burden, we repeated the experiment using CD1d-deficient mice as recipients, a setting where the presence of CD1d on the surface of ALL blasts from Eμ-ret transgenic BALB/c mice would serve as a novel antigen. In this setting, CpG ODN achieved complete protection of leukemia bearing mice (Figure 4C). Similarly, all three TLR agonists achieved significant survival improvements for recipients of primary E2A-PBX1 B-ALL cells in which GFP acts as a novel antigen (Figure A6, Appendix A) [33]. These findings indicate that each TLR treatment is capable of inducing protective immunity in the presence of a strong target antigen.

## 3. Discussion

The detection of MRD in bone marrow at the end of remission-induction therapy steers ALL patients to a risk-stratified intensification of treatment. The success of this approach at reducing the incidence of relapse confirms leukemia-involved bone marrow as a primary source of recurrent ALL subpopulations [34,35] and suggests that the application of effective strategies to further deplete these residual blasts could have significant therapeutic benefit. In this study, we reveal the differential organ-specific immune modulation achieved by endosomal TLR agonists and identify the association of leukemia depletion from the bone marrow with the most durable control of syngeneic primary B-ALL. These findings support the further investigation of immune modulation as a potential component of early therapy for children with B-ALL.

Despite the capacity of each TLR agonist to induce an early reduction in leukemia burden leading to extended disease-free survival, systemically administered CpG ODN conferred the most significant enhancement of long-term control of B-ALL. This durable protection was in accord with the superiority of CpG ODN under our experimental conditions for activating innate immune cells in a tissue-specific manner, as well as its ability to induce a rapid production of pro-inflammatory cytokines. Although three doses of polyI:C or R848 reduced B-ALL burden in bone marrow, neither achieved the magnitude of early innate immune responses or the near-complete elimination of leukemic cells from the niche observed following CpG ODN treatment. The intra-group variation in response, especially to R848 and polyI:C, suggests that biological variables within individuals (such as differences between individual B-ALL samples, leukemia burden and localisation, and immune status) may influence outcome. However, the general failure of polyI:C- and R848-treated mice to achieve prolonged survival despite the effective depletion of B-ALL cells from spleen and peripheral blood implicates the induction of immune activity in the bone marrow as a key requirement for sustained protection.

As durable long-term protection is mediated by T cell responses in our model [27], the observed differences between CpG ODN- and R848- or polyI:C-induced innate immune responses imply the superior ability of CpG ODN to induce productive T cell-priming. The activation and functional maturation of APCs are essential for promoting inflammatory responses and propagating host immune effector functions, as well as initiating adaptive immune responses. The upregulation of MHC class II expression on classically activated macrophages is IFN-γ-dependent and promotes Th1 responses by eliciting a prompt release of IL-12 [36]. In addition to their cytotoxic role, activated NK cells can provide an early source of IFN-γ necessary for the subsequent induction of Th1-polarized responses [37]. The robust and rapid activation of NK cells, in accord with the early detection of IFN-γ secretion, support their critical role in anti-ALL immune activity induced by CpG ODN. In both the syngeneic and xenogeneic settings, NK cells are a contributor to TLR-induced early immune-mediated depletion of B-ALL cells [16]. The prolonged upregulation of MHC class II, coupled with upregulated expression of co-stimulatory molecules, on macrophages in bone marrow following CpG ODN treatment may be the result of sufficient NK-mediated IFN-γ production for optimal macrophage-driven Th1-polarization. Furthermore, the significant increase in the number of cDCs following CpG ODN treatment may represent the accumulation of mature DCs with migratory capacity capable of priming T cells required for establishing long-term protection against ALL. Notably, the cell populations implicated in the TLR-induced control of primary B-ALL in this study are present early in treatment at numbers that correlate with ALC [13].

The low mutation burden and poor antigen presentation capacity of ALL blasts, in concert with a rapid induction of T cell dysfunction by progressive leukemia may contribute to the failure of immune therapy in many ALL patients [22,24,28,29,38]. Our results indicate that TLR agonists, in particular CpG ODN, can exert sufficient immunomodulatory activity to overcome these inhibitory mechanisms to eliminate primary B-ALL cells in the absence of a strong rejection antigen. The ability of TLR agonists to induce such protective immune activity in mice receiving conventional ALL chemotherapy drugs should now be assessed. Furthermore, given the ability of CTLA4 blockade to prolong survival of Eμ-ret mice [39], the superior immunostimulatory ability of CpG ODN to elicit durable protection against leukemia outgrowth demonstrated in this study provides additional support for investigating checkpoint inhibitors in combination with TLR agonists to maximize therapeutic efficacy. Such productive immune-stimulatory approaches may also enhance epitope spreading after CAR-T therapy, a phenomenon that may contribute to the maintenance of B-ALL remissions [39].

## 4. Materials and Methods

### 4.1. Ethics Statement

All experiments were performed in accordance with the Canadian Council of Animal Care and a University of British Columbia Animal Care Committee-approved protocol (A15-0187).

### 4.2. Mice

Wild-type, CD1d-deficient (CD1d^−/−^), and Rag-1-deficient (RAG1^−/−^) BALB/c, NOD/SCID (NOD/LtSz-scid/scid), and wild-type C57BL/6 mice were originally purchased from The Jackson Laboratory and maintained as in-house breeding colonies. Eμ-ret mice (on BALB/c background) were generously provided by Dr. Stephan Grupp (University of Pennsylvania, Philadelphia, PA, USA) and maintained through in-house breeding with purchased BALB/c females.

### 4.3. Cells

Primary B-ALL cells were harvested from the spleens of overtly leukemic Eμ-ret mice. Leukemia-involved spleens were processed with Tris-buffered ammonium chloride (TAC; pH: 7.2) to lyse red blood cells. The characteristic Eμ-ret B cell precursor leukemic cell phenotype (B220^int^/BP-1^hi^) was used to identify and quantify leukemic cell populations in all cases by flow cytometry [39]. Leukemic *E2A-PBX1* cells (GFP^+^/B220^int^/BP-1^hi^) were derived from bone marrow samples isolated from overtly leukemic E2A-PBX1-transgenic C57BL/6 mice [34]. Stably transduced GFP and firefly luciferase (GFP/luc)-expressing primary human B-ALL cells (96-ALL-GFP/luc and IR812-GFP/luc) were generated as previously described [33] and generously provided by Dr. David Barrett, University of Pennsylvania, PA, USA.

### 4.4. PCR

RNA isolated from mouse primary B-ALL cells using a RNeasy^®^ Plus mini kit (Qiagen, Hilden, Germany) and qScript cDNA SuperMix (Quanta Biosciences, Beverly, MA, USA) was used for first strand synthesis. Subsequent PCR was performed using *Taq* polymerase (New England Biolabs, Ipswich, MA, USA). The TLR-specific primers used were

*Tlr3*F—TCGGATTCTTGGTTTCAAGG; *Tlr3*R—TTTCGGCTTCTTTTGATGCT;
*Tlr7*F—GGAGCTCTGTCCTTGAGTGG; *Tlr7*R—CAAGGCATGTCCTAGGTGGT;
*Tlr8*F—GGCACAACTCCCTTGTGATT; *Tlr8*R—CATTTGGGTGCTGTTGTTTG;
*Tlr9*F—TCGCTTTGTGGACTTGTCAG; *Tlr9*R—GGCTCAGGCTAAGACACTGG.

### 4.5. Adoptive Transfer Experiments

Mouse or human primary leukemia cells (1 × 10^5^ cells) were adoptively transferred into 4–6-week-old wild-type, RAG1^−/−^, or CD1d^−/−^ BALB/c mice, or wild-type C57BL/6 or NOS/SCID mice by injection into lateral tail vein. Dose selection for CpG ODN (100 μg) was based on published protocols [18,27]. No significant difference in efficacy was observed with intraperitoneal (ip) and intravenous (iv) injection of TLR agonists (Figure A5, Appendix A), so ip injection was used throughout the study to avoid potential difficulties associated with repeated tail vein injections. Starting on day 7, mice were injected ip with CpG ODN (1826), R848, or polyI:C HMW VacciGrade™ (all from InvivoGen, San Diego, CA, USA) in 200 μL PBS once or with 3 doses 4 days apart. Following single-dose experiments, mice were sacrificed 3 days after treatment for evaluation of disease burden in spleen and bone marrow. For 3-dose experiments, mice were either monitored for leukemia onset, defined by hindleg paralysis, palpable lymph nodes, or white blood cell counts of >15,000/μL, or sacrificed at day 21 (6 days after the last treatment) for evaluation of disease burden in peripheral blood, spleen, and bone marrow by flow cytometry.

### 4.6. Flow Cytometry

To minimize non-specific binding of antibodies to FcγR, cells were pre-incubated with anti-mouse CD16/32 (93; BioLegend) prior to performing surface staining. Immune cell subsets were defined based on following surface phenotypes: F4/80^+^CD11b^+^ CD11c^−^ (macrophages); CD335^+^ (NK cells); and CD11b^+^CD11c^+^B220^−^MHC-II^+^ (CD11b^+^ classical DC (cDC)). All cells were stained with 7-AAD (BioLegend) to exclude dead cells. CountBright beads (Invitrogen) were used to calculate absolute cell numbers by flow cytometry. Sample collection was performed on a BD Fortessa X-20 cytometer and data analyzed using FlowJo V.10.1r7 (Treestar, Ashland, OR, USA).

### 4.7. In Vivo Bioluminescence Imaging

Human primary leukemia cells (96-ALL-GFP/luc or IR812-GPF/luc) were injected via tail vein into 4–6 weeks old NOD/SCID mice. For imaging, mice were injected with D-luciferin (GoldBio) intraperitoneally 5 min prior to imaging. Between day 21 and 24, when the average systemic radiance had reached >1 × 10^7^ p/scm^2^, mice were injected with 3 doses of the indicated TLR agonist or PBS over 8 days. Disease burden was monitored using bioluminescence, with an experimental endpoint of radiance >1 × 10^9^ p/scm^2^. Change in disease burden was determined by measuring the difference in bioluminescence immediately prior to the first treatment and 4 days after the last treatment. All live imaging was performed on an Ami-X (Spectral Instruments Imaging, Tucson, AZ, USA) and analyzed using AMIView (Spectral Insturments Imaging).

### 4.8. Serum Cytokine Analysis

Serum collected from wild-type mice 16 h after a single TLR treatment was stored at −80 °C until analysis for pro-inflammatory cytokines. Serum concentration of TNF-α, IL-6, IFN-γ, and IL-12p70 were measured with the MDS “V-Plex Custom Proinflammatory Panel 1” (Meso Scale Discovery, Rockville, MD, USA), according to manufacturer’s instructions. Serum concentration of glutamate dehydrogenase (GDH/GLHD) was measured with the GDH/GLDH ELSIA kit (Elabscience, Houston, TX, USA), according to manufacturer’s instructions. In all cases, serum was removed from the clotted sample within 1 h of blood collection.

### 4.9. Statistical Methods

Analyses of leukemic cell burden and immune cell activation in adoptive transfer experiments were performed using a one-way ANOVA with Dunn’s multiple comparisons tests for post-hoc comparisons in any case where more than two groups were being compared. Two-way ANOVA with Tukey’s multiple comparisons post-hoc tests were used when two variables were compared. Kaplan-Meier curves generated for leukemic cell adoptive transfer survival studies were analyzed by log-rank tests. Statistical analyses were performed using Prism 5 for Mac OS X (GraphPad Software Inc, San Diego, CA, USA). Specific n values for each experiment are listed in figure legends.

## 5. Conclusions

The ability of TLR-induced innate immune responses to drive adaptive immunity offers considerable potential as an approach for cancer immunotherapy. Our results from both human and mouse B-ALL models indicate that although each endosomal TLR agonist tested can achieve an early reduction in leukemia burden, systemic administration of CpG ODN confers the most significant disease-free survival benefit. Notably, CpG ODN induces a near-complete elimination of B-ALL cells from the bone marrow, the niche that most often harbours the treatment-resistant leukemic blasts that will give rise to relapse. Overall, our findings identify key components of a durable TLR-induced protective anti-leukemia response and implicate bone marrow as a potentially key target site for establishing durable remission of ALL.
